# Variations in mitragynine content in the naturally growing Kratom (*Mitragyna speciosa*) population of Thailand

**DOI:** 10.3389/fpls.2022.1028547

**Published:** 2022-10-27

**Authors:** Nisa Leksungnoen, Tushar Andriyas, Chatchai Ngernsaengsaruay, Suwimon Uthairatsamee, Phruet Racharak, Weerasin Sonjaroon, Roger Kjelgren, Brian J. Pearson, Christopher R. McCurdy, Abhisheak Sharma

**Affiliations:** ^1^ Department of Forest Biology, Faculty of Forestry, Kasetsart University, Bangkok, Thailand; ^2^ Kasetsart University Research and Development Institute (KURDI), Kasetsart University, Bangkok, Thailand; ^3^ Center for Advance Studies in Tropical Natural Resources, National Research University-Kasetsart University, Kasetsart University, Bangkok, Thailand; ^4^ Department of Botany, Faculty of Science, Kasetsart University, Bangkok, Thailand; ^5^ School of Integrated Science, Kasetsart University, Bangkok, Thailand; ^6^ The University of Florida (UF)/Institute of Food and Agricultural Sciences (IFAS) Department of Environmental Horticulture, Mid-Florida Research and Education Center, Institute of Food and Agricultural Sciences, University of Florida, Apopka, FL, United States; ^7^ Department of Medicinal Chemistry, College of Pharmacy, University of Florida, Gainesville, FL, United States; ^8^ Department of Pharmaceutics, University of Florida, Gainesville, FL, United States

**Keywords:** *Mitragyna speciosa*, kratom, mitragynine, canonical correspondence analysis (CCA), environmental factors

## Abstract

We analyzed the content of mitragynine (MG) found in kratom leaves (*Mitragyna speciosa*) and the influence of different environmental conditions (air and soil variables) on the yield in various regions of Thailand. The content of MG in kratom leaves ranged from 7.5 – 26.6 mg g^-1^ of dry leaf weight. Canonical correspondence analysis showed that the most significant environmental variables affecting the MG content among the various regions were light intensity, relative humidity, soil volumetric water content (VW), soil pH, and calcium. This study is a first step towards providing information about environmental conditions suitable to maximize the quality and quantity of bioactive alkaloids in kratom. Future studies should focus on leaf collection and the post-harvest processes in order to assure the desired alkaloidal content in finished products, when produced under suitable environmental conditions identified in this study.

## Introduction

Kratom (*Mitragyna speciosa*) is in a small Afro-Asian genus characterized by mitriform stipules at the base of the leaf and globular flowering heads (Razafimandimbison and Bremer, 2002). The species itself is a tropical, facultative deciduous, small to medium sized (4–16 m) tree, native to peninsular Thailand, Malaysia, and other countries in tropical Southeast Asia (Eisenman, 2014) and has also been reported in Vietnam and Myanmar (Suwanlert, 1975). Its leaves contain secondary metabolites (SMs) and are consumed either soaked in tea or chewed by natives and laborers for its euphoric effects at low doses (Babu et al., 2008). Kratom has long been used in traditional medicine in Southeast Asia to treat diarrhea, fatigue, cough, hypertension, and as an analgesic (Suwanlert, 1975; Cinosi et al., 2015; Grundmann, 2017).

Opioid analgesics (e.g., morphine, fentanyl, and oxycodone) are the most widely prescribed medications in the U.S. (Smith et al., 2022) and their misuse has massively increased over recent decades. The cost of the opioid-related overdose epidemic in the U.S.A. was estimated at $80 billion annually (Volkow et al., 2018). The current global attention on kratom is mainly due to its potential as an opioid-replacement medication to alleviate the withdrawal symptoms (Hassan et al., 2020) and to relieve anxiety (Hazim et al., 2014). Even though previously illegal in many countries, kratom is gaining acceptance as a medicinal plant by law in Thailand. Kratom contains a number of indole alkaloids believed to have psychoactive effects, with both the methanolic and crude alkaloid extracts demonstrating analgesic properties in rodents (Kruegel and Grundmann, 2018) and in humans, as reported in recent randomized controlled trials (Vicknasingam et al., 2020).

Alkaloids are a class of SMs, initiated through the biosynthesis of basic glycolysis or shikimic acid pathways, with a subsequent diversification largely dependent on cell type, developmental stage, and environmental cues (Patra et al., 2013). Such compounds are widely distributed in various parts of a plant such as cells, tissues, and organs. To date, several structurally related alkaloids, flavonoids, polyphenols, and terpenoid saponins have also been reported in kratom (Ramanathan et al., 2021), with alkaloids being of primary interest. Notable alkaloids include mitragynine (MG), speciogynine, speciociliatine, paynantheine, mitraphylline, and ajmalicine (Takayama, 2004; Kruegel and Grundmann, 2018; Kamble et al., 2022) and have shown potential therapeutic activities through various central nervous system (CNS) targets (Lydecker et al., 2016; Wilson et al., 2020; Sharma and McCurdy, 2021). Among these, MG has been the focus of several studies, as it is found in the largest content (Sharma and McCurdy, 2021; Zhang et al., 2022).

The production and accumulation of SMs can be modulated according to biotic and abiotic variability as a survival response to environmental stimuli (Metlen et al., 2009; Ma et al., 2010), protecting the plant from disease and environmental stresses (Li et al., 2012; Li et al., 2013), through adaptation of morphological, anatomical, and physiological functions. Most alkaloids form in the younger tissue when the plant is more susceptible to environmental changes and stresses (Houghton et al., 1991). Alkaloid content also depends on geographic location, climate, environmental demand, soil type, and soil composition (Chear et al., 2021). Kratom sampled from the South-Central U.S. has been reported to have 7-fold lower MG content than the Southeast Asian samples (León et al., 2009; Gogineni et al., 2019). Matsumoto et al. (2005) and Takayama et al. (1998) reported higher MG content in trees growing in Thailand compared to Malaysia, with its levels varying from 66% (as a fraction of total alkaloids) in trees of Thai origin (Ponglux et al., 1994) against 12% in trees of Malaysian origin (Takayama et al., 1998). There is ongoing uncertainty regarding the optimum environmental conditions for the growth and alkaloid production in kratom. The endeavor of this study was thus to determine what environmental factors influenced the variability in alkaloid content, in particular MG, in various regions of Thailand, using constrained ordination analysis.

## Materials and methods

### Plant materials

**Figure 1:**
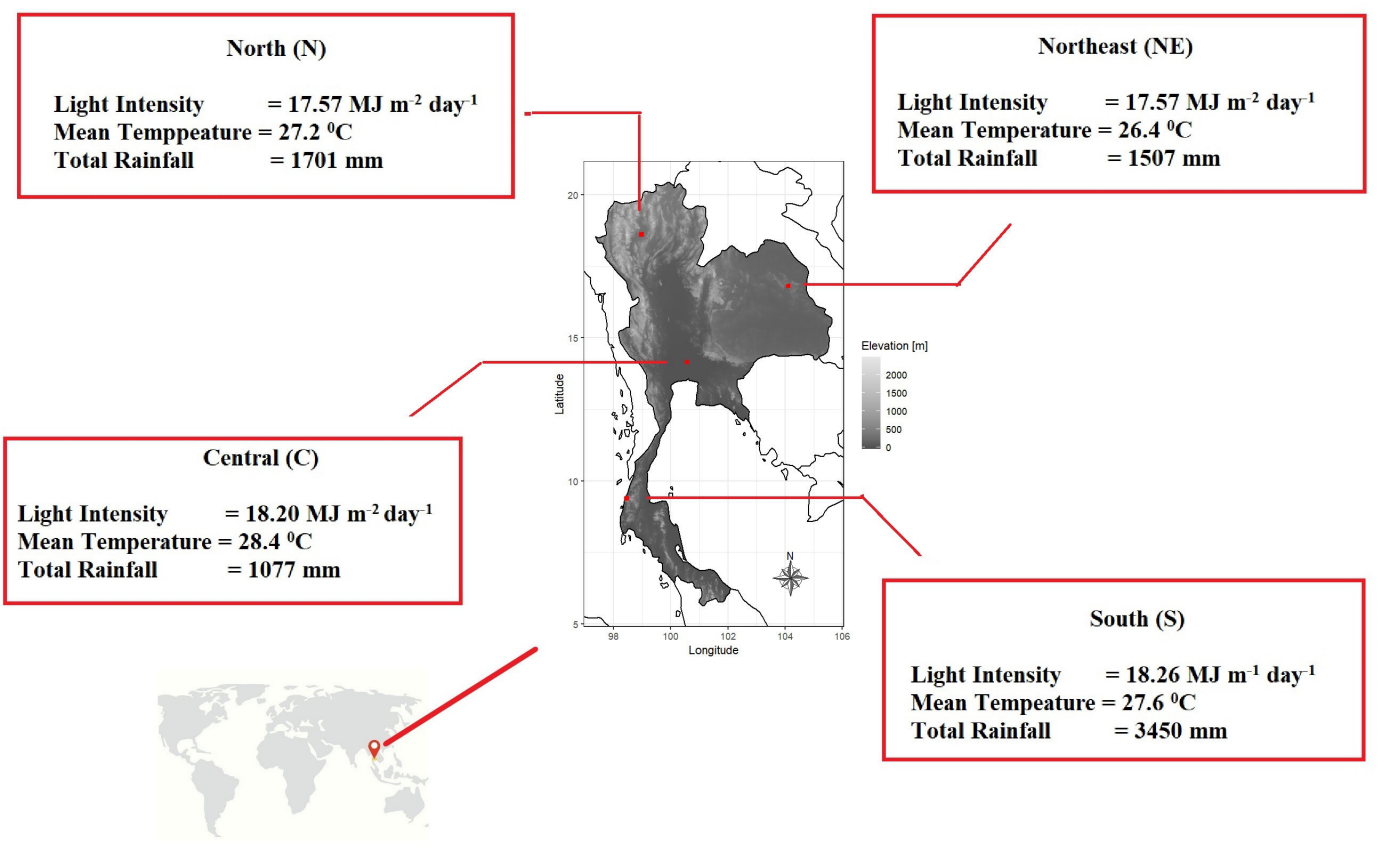
Kratom trees (*Mitragyna speciosa*) randomly collected from four regions of Thailand including north, northeast, central, and south with the description of average microclimate air variables in each region during a 30-year period (1991 – 2020) obtained from the Department of Meteorology.

*Mitragyna speciosa* (kratom) is native to the southern part of Thailand, which has a peninsular/coastal topography with high rainfall, short dry season, and limited monsoonal seasonality. The air temperature in this region is generally moderate throughout the year compared to other parts of the country with a mean air temperature between 27.0 – 28.4°C. However, kratom has also been planted successfully in other parts of the country for medicinal usage. We conducted a survey of kratom trees growing naturally on private properties in four distinct regions of Thailand including south (S), north (N), central (C), and northeast (NE) (**Figure 1** and **Table 1**; for more information visit the website http://physio.forest.ku.ac.th/mitragyna). Although the exact age of trees was unknown (as kratom does not produce annual rings which can be used to determine its age), it was approximated between 5 – 25 years, based on owner’s feedback. This wide distribution of age can be attributed to the narcotics law that banned the growing of kratom until recently (August, 2021, when the ban was removed), which has subsequently allowed it to be planted across Thailand. Hence, the samples were collected between September to December, 2021.

**Table 1:** Kratom (*Mitragyna speciosa*) samples collected from four locations across Thailand.

No.	Province (Part)	Specimen names	Code name	Collection Coordinates (Latitude and Longitude)		Elevation (m)	Tree characteristics		
				X	Y		Circumference (cm)	Height (m)	Crown area (m^2^)
1	Ayutthaya (Central)	*Ngernsaengsaruay et al. Ms01-26092021*	MS1	670341	1565337	12.0	57.0	7.7	28.7
2	Ayutthaya (Central)	*Ngernsaengsaruay et al. Ms02-26092021*	MS2	670372	1565341	5.0	22.0	4.0	13.9
3	Ayutthaya (Central)	*Ngernsaengsaruay et al. Ms03-26092021*	MS3	670314	1565344	2.0	51.0	7.7	15.6
4	Ayutthaya (Central)	*Ngernsaengsaruay et al. Ms04-26092021*	MS4	670106	1565114	11.0	54.0	4.0	11.8
5	Ayutthaya (Central)	*Ngernsaengsaruay et al. Ms05-26092021*	MS5	670350	1565320	10.0	74.2	8.0	21.9
6	Ranong (South)	*Ngernsaengsaruay et al. Ms06-21102021*	MS6	440938	1041507	0.0	61.0	10.7	30.7
7	Ranong (South)	*Ngernsaengsaruay et al. Ms07-21102021*	MS7	439794	1039142	0.0	92.0	14.0	41.8
8	Ranong (South)	*Ngernsaengsaruay et al. Ms08-21102021*	MS8	440806	1036798	31.0	85.0	14.5	12.9
9	Ranong (South)	*Ngernsaengsaruay et al. Ms09-21102021*	MS9	441830	1036749	39.0	31.5	9.7	15.9
10	Ranong (South)	*Ngernsaengsaruay et al. Ms10-21102021*	MS10	439640	1038369	30.0	82.0	15.0	40.7
11	Mukdahan (Northeast)	*Ngernsaengsaruay et al. Ms11-24112021*	MS11	405291	1859013	317.0	25.3	5.0	15.9
12	Mukdahan (Northeast)	*Ngernsaengsaruay et al. Ms12-24112021*	MS12	405287	1859014	321.0	16.0	5.1	2.7
13	Mukdahan (Northeast)	*Ngernsaengsaruay et al. Ms13-24112021*	MS13	405295	1859007	319.0	15.8	4.0	7.8
14	Mukdahan (Northeast)	*Ngernsaengsaruay et al. Ms14-24112021*	MS14	405269	1859029	309.0	15.5	4.0	3.0
15	Mukdahan (Northeast)	*Ngernsaengsaruay et al. Ms15-24112021*	MS15	405278	1859024	304.0	18.0	5.0	5.5
16	Lamphun (North)	*Ngernsaengsaruay et al. Ms16-18122021*	MS16	496848	2057407	292.0	22.0	6.0	8.1
									
17	Lamphun (North)	*Ngernsaengsaruay et al. Ms17-18122021*	MS17	498869	2057325	303.0	35.0	7.0	12.5
18	Lamphun (North)	*Ngernsaengsaruay et al. Ms18-18122021*	MS18	498826	2059233	290.0	40.0	3.3	15.4
19	Chiang Mai (North)	*Ngernsaengsaruay et al. Ms19-18122021*	MS19	493746	2113200	361.0	14.5	5.2	3.8

In each region, 4 - 5 kratom trees were chosen based on the recommendations listed in the handbook for functional traits by Perez-Harguindeguy et al. (2016) (see **Figure 1** and **Table 1**). The species identification was conducted by Chatchai Ngernsangsaruay, Associate Professor in the Department of Botany, Faculty of Science, Kasetsart University, following the recommended international guidelines for sample collection and identification in the Flora of Thailand (Chayamarit and Balslev, 2021; Ngernsaengsaruay et al., 2022). Kratom voucher specimens were compared with the specimens maintained in the Bangkok Forest Herbarium (BFK), Royal Forest Department, Bangkok, Thailand and were stored at the Herbarium of the Faculty of Science, Kasetsart University.

#### Measurements

Growth characteristics, leaf traits, and environmental conditions were measured using standard methods and instruments summarized in **Table 2**. As indicated in the table, growth characteristics included stem circumference, tree height, and crown area. Leaf traits included leaf area (LA), specific leaf area (SLA), leaf thickness, and leaf pH were determined in the Department of Forest Biology laboratory, Kasetsart University, within 3 days after leaf sample collection. Physiological traits included maximum photosynthesis rate (Pnmax, and P2000), chlorophyll content (SPAD), chlorophyll performance index (PI), and quantum yield (Fv/Fm) and were measured onsite. Environmental air variables in the four regions included temperature, relative humidity, rainfall, and light intensity and were obtained from the Department of Meteorology over a 30-year period (1991 – 2020). The soil characteristics including volumetric water content (VW), bulk density (BD), pH, organic matter (OM), carbon (C), nitrogen (N) phosphorus (P), potassium (K), calcium (Ca), and magnesium (Mg) were analyzed at the soil laboratory in the Department of Silviculture, Kasetsart University. The respective units are also indicated in **Table 2**.

**Table 2:** Measurements for Kratom trees in four regions of Thailand including growth, leaf traits, and environments.

Characteristics	Methods	Instruments	Sample	References
**Growth characteristics** (5 trees per region except the north with 4 trees**)**				
1. Stem Circumference (cm)	Girth at breast height (GBH) 1.30 m.	Measuring tape	–	Yarnvudhi et al., 2021
2. Tree Height (m)	Total height	rangefinder (Nikon forestry Pro, Nikon Inc., Tokyo, Japan)	–	“
3. Crown Area (m^2^)	Crown radius (r) in 2 perpendicular directions. Areas = πr^2^	Measuring tape	–	“
**Leaf traits characteristics** (5 trees per region except the north with 4 trees**)**				
4. Leaf Area (LA) (cm^2^)	Image processing	ImageJ (Rasband, W.S., ImageJ, U. S. National Institutes of Health, Bethesda, Maryland, USA)	10 leaves per tree	Perez-Harguindeguy et al., 2016 https://imagej.nih.gov/ij/
5. Specific Leaf Area (SLA) (cm^2^ g^-1^)	SLA = LA/dry mass (g)	Oven dry and 3 digits analytical balance (Precisa Model XT 620M, Precisa Gravimetrics AG, Dietikon, Switzerland)	“	Perez-Harguindeguy et al., 2016
6. Leaf Thickness (mm)	Avoid the vein of the leaf blade	Digimatic Thickness Guage (6 digits) (Model 547 Mitutoyo Cooperation, Kawasaki, Japan)	“	Pauli et al., 2017
7. Leaf pH (Unitless)	5 g. of fresh leaf sample was ground and mixed with 40 g. of deionized water (ratio 1:8) and filtered prior to being measured.	Handheld pH meter (Model PCSTestr 35, Eutech Instruments Pte Ltd, Singapore)	“	Perez-Harguindeguy et al., 2016
8. Maximum Photosynthesis Rate (P_n_max, and P_2000_) (μmol m^-2^ s^-1^)	Light response curve from 0 – 2000 μmol m^-2^ s^-1^) under controlled conditions of temperature at 30°C, RH at 60%, air flow at 500 μmol s^-1^, and CO_2_ at 400 ppm. P_n_max at any given light intensity and P_2000_ at light intensity of 2000 μmol m^-2^ s^-1^ were recorded.	Portable Photosynthesis System (Li-6800, LI-COR Corporate, Nebraska, USA)	One sunlit leaf per tree	Sonjaroon et al., 2016
9. Chlorophyll Content (SPAD)	5 spots on leaf blade were measured and averaged to be 1 value for each tree.	Chlorophyll meter (SPAD-502, Konica Minolta Sensing Europe, London, UK)	10 leaves per tree	Leksungnoen, 2017
10. Chlorophyll Performance Index (PI) (Unitless)	2 dark-adapted clips were placed on a leaf blade for 15 – 30 mins of each tree prior to take measurement. Values were then averaged to be 1 value for each tree.	Chlorophyll fluorescence meter (Handy PEA, Hansatech, Norfolk, UK)	“	Sonjaroon et al., 2016
11. Quantum Yield (Fv/Fm) (Unitless)	“	“	“	“
**Environment characteristics**				
12. Air conditions; 12.1 Temperature (°C) 12.2 Rainfall (mm) 12.3 Light Intensity (MJ m^-2^ day^-1^)	Secondary data	Weather station in proximity area of Kratom trees in each region (less than 30 km away)	30-year period (1991 – 2020)	^1^Department of Alternative Energy Development and Efficiency, Ministry of Energy www.dede.go.th ^2^Department of Meteorology, Ministry of Digital Economy and Society www.tmd.go.th
13. Soil Conditions (Laboratory test by Soil Laboratory, Department of Silviculture, Faculty of Forestry, Kasetsart University, Bangkok, Thailand)				
13.1 Volumetric Water Content (VWC) (%)	VWC = (water volume/soil volume) × 100	Soil core (5.7 cm in diameter) (Soilmoisture Equipment Corp., California, USA) at soil depth 30 – 45 cm.	3 replicates per tree	Carter and Gregorich, 2007
13.2 Bulk Density (BD) (g cm^-3^)	BD = soil dry mass/soil volume	“	“	“
13.3 Porosity (P) (%)	P = (1 - (BD/Particle Density)) x 100	Calculation	“	“
13.4 pH (Unitless)	1:1 ratio	Disturbed soil samples collected from 3 locations around the root zone at depth 0 – 45 cm.	1 replicate per tree	Sparks et al., 2020
13.5 Organic Matter 13.6 (OM) (%)	Walkley and Black	“	“	Walkley, 1947
13.7 Carbon (C) (%)	Dry combustion	“	“	Sparks et al., 2020
13.8 Nitrogen (N) (%)	Dry combustion	“	“	“
13.9 Phosphorus (P) (mg kg^-1^)	Atomic Absorption and Flame Emission Spectrometry (AAS-Flame)	“	“	“
13.10 Potassium (K) (mg kg^-1^)	“	“	“	“
13.11 Calcium (Ca) (mg kg^-1^)	Atomic Absorption and Flame Emission Spectrometry (AAS-Flame)	Disturbed soil sample collected from 3 locations around the root zone at depth 0 – 45 cm.	1 replicate per tree	“
13.12 Magnesium (Mg) (mg kg^-1^)	“	“	“	“

##### Determination of MG concentration

**Figure 2:**
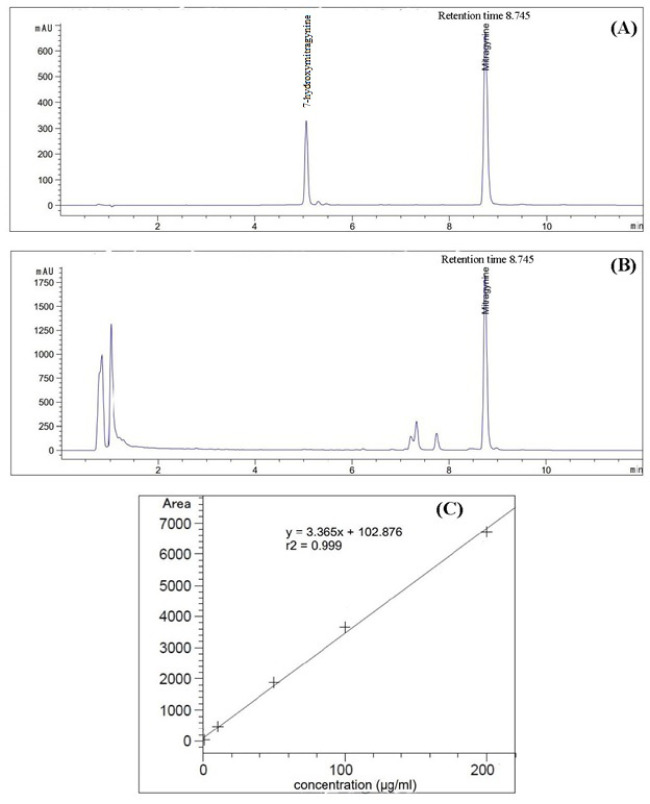
Chromatograms from High-Performance Liquid Chromatography-Diode Array Detection (HPLC-DAD) of: **(A)** MG and 7OH-MG standards; **(B)** methanolic extract of kratom sample. The standard calibration curve with equation and coefficient of determination (R^2^) **(C)**.

All the collected leaves were cleaned to remove dirt particles and placed in a hot air oven at 45 °C until they were dry and had reached a constant mass over a two-day period. The dried leaves were ground to a fine powder using a mechanical blender. The powdered dried leaves (20 g) were then soaked in 200 ml of methanol for three days at room temperature and were shaken occasionally. The mixture was then filtered with Whatman No. 1 (Merck Ltd., Darmstadt, Germany) and concentrated using a rotary vacuum evaporator (Buchi, Rotavapor^®^ R-100, Switzerland) under a reduced pressure at 40 °C. The MG content was quantified with High-Performance Liquid Chromatography-Diode Array Detection (HPLC-DAD) using the method suggested by Mudge and Brown (2017). The analysis was performed using Agilent 1260 Infinity II LC System equipped with Kinetex^®^ Evo C18 column (150 mm x 4.6 mm, 5 µm particle), a column thermostat, a vial-autosampler system, a quaternary pump with a degasser and DAD detector, with the column temperature set to 25°C and sample injection volume of 2.5 µl. LC gradient elution was done using a mobile phase consisting of ammonium bicarbonate (5 mM, pH 9.5; A) and acetonitrile (B), with the flow rate of the mobile phase being 1.5 ml/min. The initial concentration of B was set to 30% and was gradually increased to 70% for seven min, until it was held at 70% for three min. A DAD, at wavelength of 226 nm, was used for data acquisition and MG content in the crude extracts was identified by comparing the peak retention times and UV profiles with the reference MG standard. Peak purity test was also done to ensure method selectivity for a reliable quantification of MG with a purity factor >999) (**Figures 2A, B**).

A stock solution of the mixed reference MG and 7-hydroxymitragynine (7OH-MG) standards (Cayman Chemical Company, Michigan, USA) was prepared by dissolving the standard solution in HPLC grade methanol to obtain a concentration of 1 mg/ml. The stock solution was further diluted to obtain the target solution levels of 1, 10, 50, 100 and 200 µg/ml and a calibration curve was constructed subsequently. To prepare a sample solution for the analysis, the crude extracts were dissolved in HPLC grade methanol to obtain a concentration of 10 mg/ml, which was then injected into the HPLC system. The MG content (of three replicates) were calculated based on the peak area under the calibration curve of the MG standard using the LC-solution software. The calibration curve of MG was found to be linear over a concentration range of 1-200 µg/ml with an equation of the form y = 3.365x + 102.876 with the coefficient of determination (R^2^) = 0.99 (**Figure 2C**).

##### Canonical correspondence analysis (CCA)

The Canonical Correspondence Analysis (CCA) ordination was used to assess the alkaloidal variations constrained by the environmental and soil predictors. Inaccurate estimates obtained through linear analysis were avoided by using only unimodal analyses such the CCA. It is a direct gradient technique that simultaneously represents sites, environmental variables, and alkaloids in a low dimensional space (Ter Braak, 1987) and has been previously used to predict species or community distributions (Hill, 1991; Gottfried et al., 1998). The CCA axes are constrained to be linear combinations of the environmental variables, with the axes representing variables that lead to a maximum separation or variation in the samples. The mean values of weather parameters were used to perform the CCA. The relative importance of each CCA axis is indicated by its eigenvalue, which measures the extent to which a linear combination of environmental variables can explain the variations in the alkaloid content (Van Tongeren et al., 1995).

We used a forward selection to filter the most significant environmental variables according to the variance explained by the variation in alkaloid data. Their significance was tested using Monte Carlo testing with 999 unrestricted permutations and the variables with *P*-value < 0.10 were deemed significant. The environmental variables with significant effect on the alkaloidal variability were then depicted on a CCA triplot (i.e., the environmental variables, MG, and the sample locations in the four regions). The analysis was carried out using the relevant CCA functions available in the *vegan* package (Dixon, 2003; Oksanen et al., 2006) of the R statistical language (R Development Core Team, 2020).

### Statistical analysis

*One-way ANOVA* was used to compare the mean growth and leaf traits among the four regions of Thailand (**Table 2**) using R program (R Development Core Team 2020). *Post hoc* mean comparisons were tested using least square differences (LSD) using R package “agricolae” (Mendiburu, 2021).

## Results

### Leaf functional traits and mitragynine content

The measured stem size and tree height of sample trees varied widely among the four regions, from 15.0 – 92.0 cm, with an average of 42.7 ± 26.3 cm and a height of 7.4 ± 3.8 m. The kratom trees had a spherical canopy with the crown area ranging between 2.7 – 41.8 m^2^ (**Table 1**). Among the four regions, trees in the S region, which happens to be their native habitat and hence treated as the control in this study, were the largest in stem circumference, height, and crown area followed by those in the C region, while the trees in N and NE regions were the smallest (**Table 3**). Among the eight leaf functional traits, half had statistically different mean values across the four regions (**Table 3**), including SLA, P2000, PI, and Fv/Fm. P2000 directly indicates the photosynthesis rate and growth and was the highest in the C and N regions, while it was lower in the S and NE regions.

**Table 3:** Comparison of mean growth, leaf traits, crude percentage, and mitragynine (MG) content among kratom trees found in 4 regions of Thailand.

Characteristics/Parts of thailand	South (Control)(S)	Central(C)	North(N)	Northeast(NE)	P-value
1. Stem Circumference (cm)	70.30 ± 24.57 a	51.64 ± 18.86 a	27.88 ± 11.70 b	18.12 ± 4.13 b	<0.0001***
2. Tree Height (m)	12.78 ± 2.41 a	6.28 ± 2.08 b	5.38 ± 1.57 b	4.62 ± 0.57 b	<0.0001***
3. Crown Area (m^2^)	28.40 ± 13.53 a	18.38 ± 6.89 ab	9.95 ± 5.08 b	6.98 ± 5.40 b	0.003**
4. Leaf Area (LA) (cm^2^)	110.41 ± 26.43	107.10 ± 26.29	109.71 ± 18.80	100.57 ± 20.51	0.911 (NS)
5. Specific Leaf Area (SLA) (cm^2^ g^-1^)	161.83 ± 23.45 a	152.89 ± 14.12 a	123.35 ± 18.12 b	165.32 ± 16.43 a	0.037*
6. Leaf Thickness (mm)	0.160 ± 0.006	0.162 ± 0.016	0.171 ± 0.010	0.174 ± 0.006	0.185 (NS)
7. Leaf pH (Unitless)	4.34 ± 0.25	4.44 ± 0.14	4.38 ± 0.32	4.72 ± 0.11	0.059 (NS)
8. Maximum Photosynthesis Rate (P_n_max) (μmol m^-2^ s^-1^)	8.10 ± 2.24	12.56 ± 4.61	12.97 ± 2.25	8.02 ± 2.43	0.051 (NS)
9. Maximum Photosynthesis Rate (P_2000_) (μmol m^-2^ s^-1^)	10.25 ± 2.55 b	16.04 ± 3.67 a	15.93 ± 2.93 a	10.01 ± 2.81 b	0.017*
10. Chlorophyll Content (SPAD)	33.79 ± 3.53	31.66 ± 2.51	33.80 ± 3.31	33.35 ± 3.48	0.700 (NS)
11. Chlorophyll Performance Index (PI) (Unitless)	3.26 ± 1.24 a	2.36 ± 0.32 a	2.41 ± 0.03 a	1.54 ± 0.38 b	0.001**
12. Quantum Yield (Fv/Fm) (Unitless)	0.80 ± 0.01 a	0.80 ± 0.01 a	0.78 ± 0.01 b	0.72 ± 0.02 c	<0.0001***
13. Crude percentage	30.10 ± 2.94	26.80 ± 3.00	29.08 ± 3.24	29.14 ± 4.31	0.499 (NS)
14. Mitragynine (MG) (mg g^-1^ dry weigh)	18.46 ± 3.31 a	21.84 ± 4.10 a	11.89 ± 4.28 b	11.14 ± 2.33 b	<0.0001***

* 0.05, ** 0.001, *** <0.001, and NS indicates not significant.

Value in the column is mean ± standard deviation (n= 4 - 5). The lowercase letters in each row indicate the statistically significant difference in mean among regions at the significant level of 95%.

We found that the SLA was significantly greater in C, S, and NE regions than trees in the N region. PI and Fv/Fm indicate the ability of chlorophyll to absorb light and pass on electrons during the light reaction in photosynthesis, with a high value indicating a chance for higher growth. Kratom trees in S and C regions had a higher PI and Fv/Fm when compared to those in N and NE. Leaf area, leaf thickness, leaf pH, and chlorophyll content were not statistically different (*P*-value < 0.05) among trees in all regions (**Table 3**), indicating that these traits were less sensitive to varying environmental conditions compared to traits related to photosynthesis and chlorophyll efficiency discussed above.

Methanol crude extraction yield was not statistically different when compared among regions (**Table 3**), ranging between 24.3 – 36.1%. The fraction of MG to total alkaloid of the MS sampled from the four regions ranged from 27.7% (MS19: Northern region) to 69.4% (MS2: Central region) within a profile of 116 identified chemicals. MG content varied widely, between 7.5 – 26.6 mg g^-1^ of dry leaf weight, with an average of 16.0 ± 5.7 mg g^-1^. The highest levels were found in C and S regions (18.5 – 21.8 mg g^-1^ dry leaf weight), while locations in the N and NE had lower levels of around 11.4 mg g^-1^.

### Canonical correspondence analysis (CCA)

**Figure 3:**
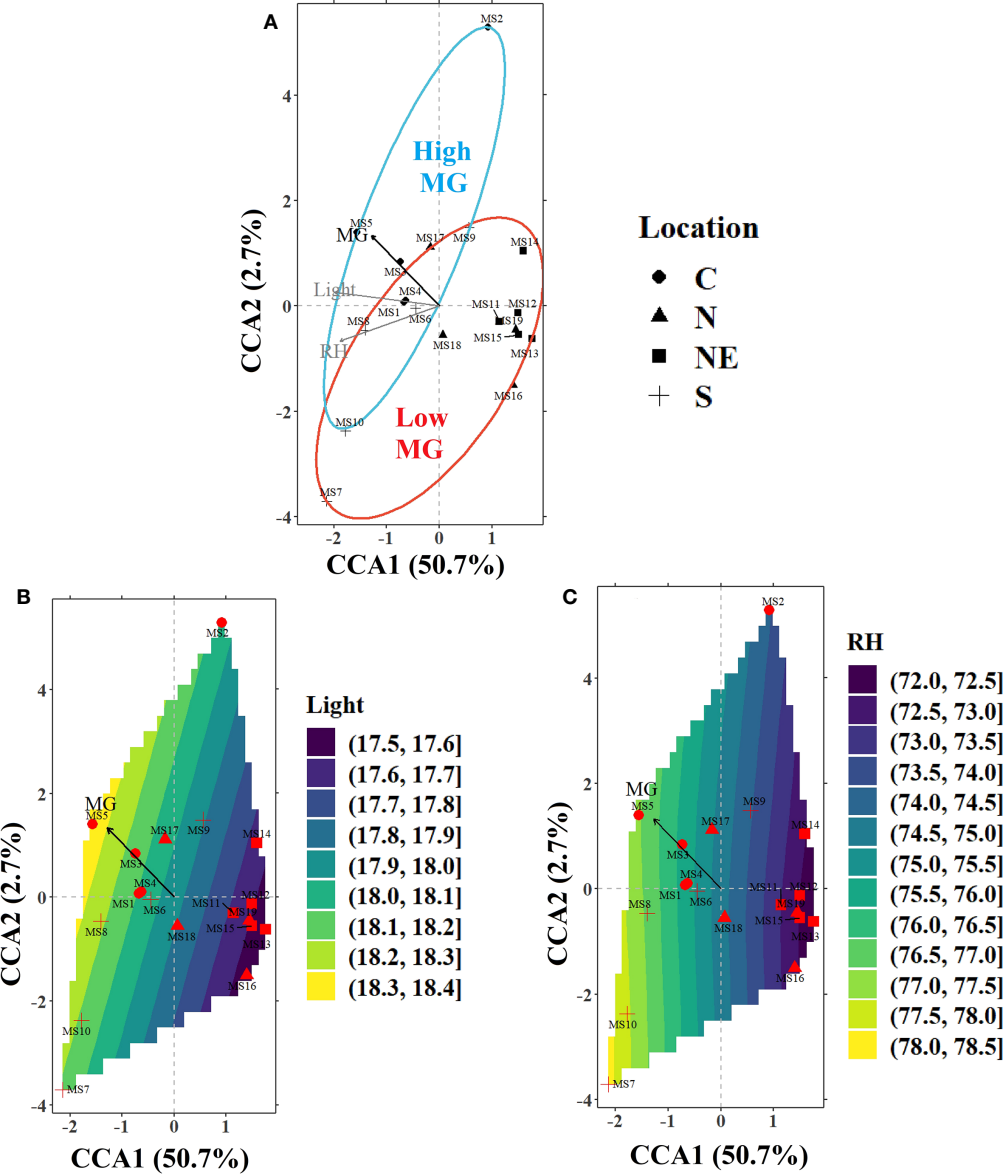
**(A)** CCA ordination plot for the significant environmental variables in the air sub-model (light intensity; light and relative humidity; RH) influencing the alkaloid levels in the sampled trees from various locations around Thailand indicated by different markers (filled circles-C, filled triangles-N, filled squares-NE, and plus sign-S). The ellipses represent 90% confidence levels for content of MG (blue for high MG and red for low MG). The contours for the environmental variables in the bottom panels represent the landscape gradients as calculated by the *ordisurf* function in vegan library of R for **(B)** light (MJ m^-2^ day^-1^) and **(C)** RH (%).

**Figure 4:**
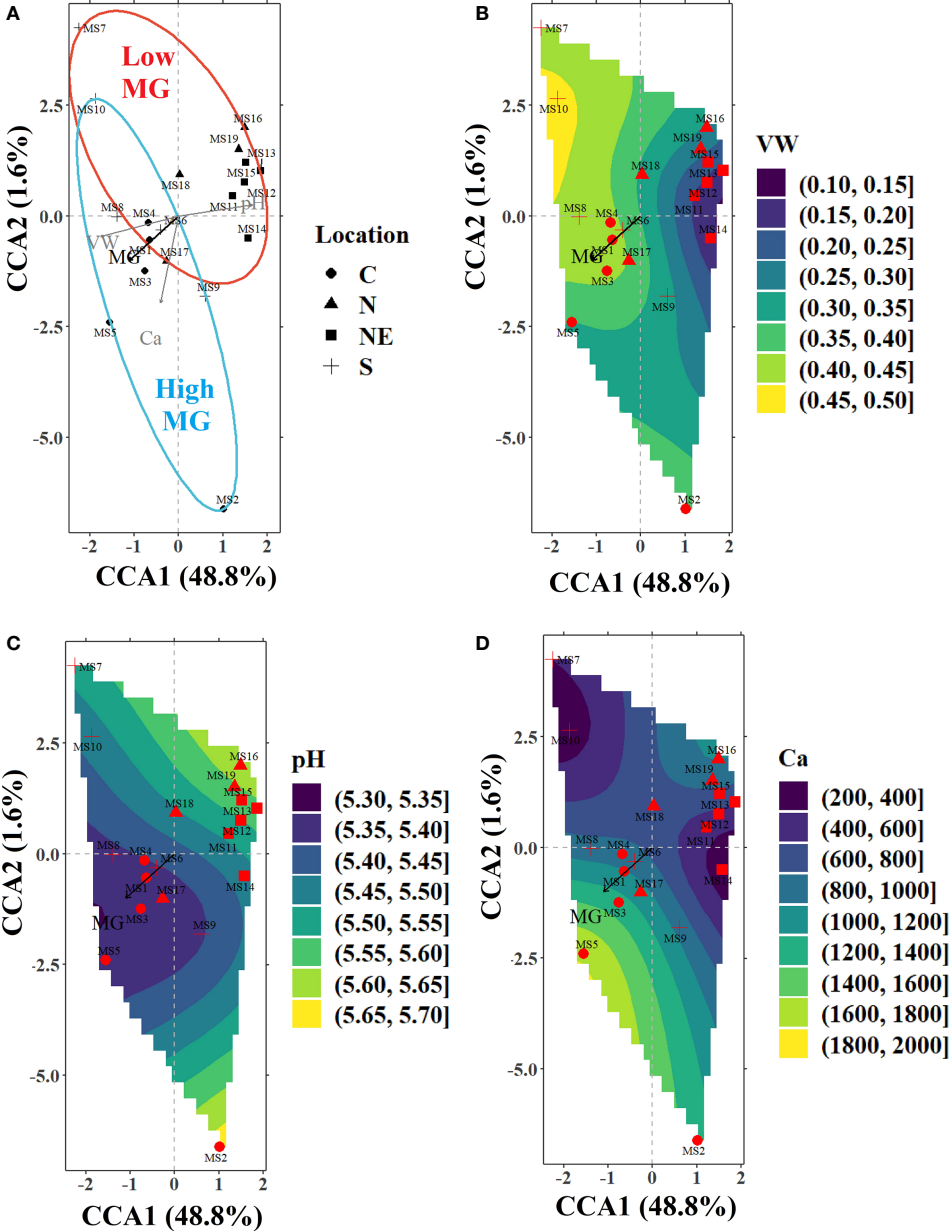
**(A)** CCA ordination plot for the significant environmental variables in the soil sub-model (volumetric water content; VW, calcium; Ca, and pH) influencing the alkaloid levels in the sampled trees from various locations indicated by different markers (filled circles-C, filled triangles-N, filled squares-NE, and plus sign-S). The ellipses represent 90% confidence levels for content of MG (blue for high MG and red for low MG). The filled contours in the bottom panels represent the nutrient gradient landscapes as calculated using the *ordisurf* function in vegan library of R for **(B)** VW (%) **(C)** pH, and **(D)** Ca (mg kg^-1^).

CCA was used to analyze the effect of environmental variables on the MG content at various sample locations in the four regions. It was observed that the initial model constructed with all the measured air (average temperature; T, light intensity; light, rainfall, and relative humidity; RH) and soil (volumetric water content; VW, bulk density, pH, organic matter; OM, carbon; C, nitrogen; N, phosphorus; P, potassium: K, calcium; Ca, and magnesium; Mg) variables was not significant (*P*-value = 0.144). However, when the air and soil variables were modeled separately, both the sub-models were found to be highly significant (air sub-model; *P*-value = 0.001 and soil sub-model; *P*-value = 0.004). Therefore, a separate analysis was undertaken for both the sub-models. After removing the collinear variables using the *vif* function in R program, a forward selection indicated that two out of the four air (light and RH) and three out of the ten soil (VW, pH, and Ca) variables significantly influenced the alkaloid levels. The most significant variables were light (air sub-model) and VW (soil sub-model), which contributed to around 38% and 42% of the total variance explained in the respective sub-models. CCA ordination for the air sub-model (see **Figure 3**) and soil sub-model (**Figure 4**) shows the relative importance of the significant variables in explaining the variance in alkaloid data and are tabulated in **Table 4**.

**Table 4:** Canonical correspondence analysis (CCA) of environmental variables (air and soil).

Environmental Variable	Marginal Effect(%; *P*-value)	Conditional Effect (%; *P*-value) [forward selection]	Pure Effect(%; *P*-value)
**Air sub-model**			
1. Tav (^°^C)	36.44 (0.004^**^)	2.32 (0.518^ns^)	-
2. Light (MJ m^-2^ day^-1^)	49.83 (0.001^***^)	37.1 (0.001^***^)	38.4 (0.001^***^)
3. Rainfall (mm)	18.86 (0.04^*^)	5.46 (0.204^ns^)	–
4. Relative Humidity (RH) (%)	45.31 (0.001^***^)	1.02 (0.046^*^)	15.03 (0.005^**^)
**Soil sub-model**			
1. Volumetric soil moisture (VW) (%)	37.9 (0.003^**^)	24.3 (0.007^**^)	41.9 (0.001^***^)
2. Bulk density (g cm^-3^)	1.10 (0.820^ns^)	7.9 (0.137^ns^)	
3. pH (Unitless)	9.95 (0.167^ns^)	**10.2 (0.081°)**	0.81 (0.479^ns^)
4. Organic matter (OM) (%)	2.50 (0.573^ns^)	1.42 (0.688^ns^)	
5. Carbon (C) (%)	0.51 (0.927^ns^)	2.01 (0.596^ns^)	
6. Nitrogen (N) (%)	0.01 (0.996^ns^)	6.03 (0.220^ns^)	
7. Phosphorus (P) (mg kg^-1^)	2.96 (0.597^ns^)	-0.22 (0.972^ns^)	-
8. Potassium (K) (mg kg^-1^)	2.70 (0.620^ns^)	4.1 (0.349^ns^)	
9. Calcium (Ca) (mg kg^-1^)	3.52 (0.497^ns^)	**10.8 (0.068°)**	7.93 (0.001^***^)
10. Magnesium (Mg) (mg kg^-1^)	2.70 (0.529^ns^)	5.21 (0.272^ns^)	

The results indicate the significance of variations in alkaloidal composition through the significant explanatory variables (the remaining variables were removed through forward selection analysis), using Monte Carlo permutation test (999 permutations) at the significant level of 90%.

The numbers in bold in any row indicate variables selected through forward elimination and used to constrain the CCA (ns: not significant). Environmental variables (mostly with first constrained axis for Temp and light). VW first, P with second, and Ca with both constrained axes ‘***’ 0.001, ‘**’ 0.01, ‘*’ 0.05, ‘°’ 0.1., (ns: not significant).

Air sub-model: The CCA axes of this sub-model (constrained using light and RH) (**Figure 3**) accounted for over 50% of the variance in the alkaloid data, with light (38.4%) having the strongest correlation with alkaloid content. The other significant variable was RH but it was relatively less important in explaining the alkaloid distribution. The first ordination axis was negatively correlated with both the significant variables with a higher light and RH favoring higher MG levels. Most of the trees in the C and S regions were located along an increasing light gradient (higher MG content), while those in N and NE were located on a decreasing light gradient (lower MG content). MS5 (C) and MS13 (NE) illustrate locations at opposite levels of the light gradient. As such, location MS5 has a high MG level whereas MS13 has a low MG level. Higher values of RH were observed in most of the locations in the C and S regions with the air being relatively drier in the N and NE regions.

Soil sub-model: The CCA axes of the soil sub-model (constrained using VW, pH, and Ca) accounted for over 48% of the variance in the alkaloid data. The first CCA axis was negatively correlated with VW and positively correlated with soil pH, while the second axis was negatively correlated with soil Ca (**Figure 4**). VW (41.9%) had the strongest correlation with alkaloid composition, while pH had a significant conditional effect on the alkaloid profile (10.2%) and no significant marginal (i.e., when tested as the only constraining variable) or pure (i.e., the percentage variance explained by the variable with the remaining significant variables used as co-variates) effect. On the other hand, soil Ca was not significant when tested as the only environmental variable (insignificant marginal effect) but had a significant conditional and pure effect (**Table 4**). Most sites in the C and S regions were located on higher levels of the VW gradient (along the direction of MG), while most in the N and NE regions were grouped around relatively drier soil (**Figure 3**). The soil was often found to be relatively acidic in the collection sites of C and S regions with high Ca content, while the sites in N and NE regions were mostly grouped around less acidic soil with lower levels of Ca. Kratom trees with high MG were located mostly along an increasing Ca gradient, for example, MS5 (C), while lower values of MG were measured in sites on the opposite direction (MS16 (N)).

## Discussion

Alkaloids are produced by plants to protect them from various abiotic and biotic stresses (Iwasa, 2000). In the case of kratom, we determined the levels of alkaloid MG in various geographical regions along environmental gradients in Thailand. Kratom is naturally found growing in inundated swampy freshwater areas which can cause hypoxic stress for roots immersed in water for an extended period of time (up to 2 – 6 months) (Ngernsaengsaruay et al., 2022). The content of MG in this study was in a range similar to that reported in other studies conducted in different geographical locations. Kikura-Hanajiri et al. (2009) reported that MG content available in the commercial kratom products in the U.S. ranged between 1 – 6%, while in our study, it was between 1.1 – 2.2%. Also, an *in-vitro* study reported a similar range in commercial products sold in the U.S., between 8.13 – 11.45 mg g^-1^ (7.5 – 26.6 mg g^-1^ in the present study) (Todd et al., 2020). Moreover, the MG content in kratom grown in Malaysia was also within a similar range between 9.38 – 18.85 mg g^-1^ (Chear et al., 2021). Hence, the MG content sampled from all the four regions in this study was in a comparable range reported previously. This indicates the potential of growing kratom in varying environmental conditions in Thailand without a substantial reduction in the amount of bioactive chemicals obtained.

Tree size could affect the total MG yield as Iwasa (2000) and Berkov et al. (2014) have suggested that different plant characteristics (age, size, species, and genetics) can result in variable content of SMs. We found that MG content was positively correlated (r = 0.554, *P*-value = 0.014, N = 19) with the girth at breast height (GBH), indicating that the larger trees produced a higher amount of MG per unit dry leaf weight. Berkov et al. (2014) reported that larger plants of a perennial species (*Narcisus* spp.) tended to produce higher content of SMs to protect themselves from threats such as herbivores. Several factors such as the stage of growth, species growth rate, and type of alkaloid etc. can play a crucial role in determining the levels of SMs. Therefore, a future study focusing on kratom alkaloid production relative to trees of different ages and stage of growth will be undertaken to better understand their effects on alkaloids.

Since the production of SMs through photoassimilation primarily depends on primary metabolites, some leaf traits related to growth and photosynthesis rate could be used as an indicator for high growth and MG content, which included P2000, PI, and Fv/Fm (**Table 2**). Shipley (2006) concluded that the photosynthesis rate (P2000 in this study) can be used to predict relative growth rate. Although SPAD and SLA have been reported to be highly correlated with higher MG content at the seedling stage (Zhang et al., 2022), the relationship was not significant for the mature trees sampled in our study (**Table 2**). Additionally, SLA has been reported to be positively related with growth elsewhere, with higher values indicating a greater leaf ability to absorb sunlight for photosynthesis (Arasa-Gisbert et al., 2022).

MG content depends on various factors such as plant genetics, post harvesting, crude extraction, and growth environments. Therefore, we used CCA to investigate the environmental gradients (both air and soil) that affect MG content. In this study, a higher amount of MG was observed under higher light, RH, VW, Ca, and under acidic soil (low pH) (see **Figure 3**). Zhang et al. (2022) had previously concluded that low-light conditions (~25% of full sunlight) maximized and promoted a greater accumulation of biomass and total alkaloid content (mainly MG) in kratom leaves, compared to leaves under full sunlight. However, their study was conducted on seedlings in greenhouse conditions, while the present study was conducted in the natural habitat of mature kratom trees. Thus, variability in the level of alkaloids relative to light could be a result of the growing stage (Li et al., 2020). Light requirement for kratom can also change, as the species is shade tolerant at seedling stage (Zhang et al., 2022) and light demanding when mature (as in our study), similar to another tropical species found in Thailand, rosewood (*Dalbergia cochinchinensis*) (Leksungnoen et al., 2021).

Atmospheric RH is the ratio of ambient air water vapor to that held at saturation at a given ambient temperature. Generally, the measured change in RH is due to change in saturation vapor (a function of ambient temperature), while the actual vapor content only changes slightly during the day. As the temperature increases, air can hold more water molecules resulting in a decrease in RH. In the present study, higher RH tended to favor higher levels of MG. This trend has been reported previously in *Dendrobium officinale* (Orchidaceae), whose total alkaloid content was affected by both maximum and minimum RH, with high maximum RH resulting in a higher total alkaloid content, while elevated minimum RH resulted in a lower total alkaloid content (Yuan et al., 2020). However, another study concluded that in a woody medicinal species (*Moringa oleifera*), higher amounts of alkaloids were produced under high temperatures or low RH (Alberto et al., 2019). Although elevated temperatures usually tend to increase the production of SMs (Naghiloo et al., 2012), some studies have also suggested that the levels of SMs can decrease under extremely high temperatures (Shibata et al., 1988).

In conjunction with soil temperature, VW and the amount of clay present in the soil, can affect the soil microbiome and may affect the plant alkaloid profile (Sudduth et al., 2003; Badri et al., 2013). Since kratom is native to swampy, freshwater forests characterized by soils which are inundated or saturated for almost the whole year (Ngernsaengsaruay et al., 2022), high soil water content appears to govern the MG content to a large degree. However, only a few studies have reported an increase in alkaloid levels under high soil moisture, as observed in the present study. Moreno-Pedraza et al. (2019) reported that tropane alkaloids in *Datura stramonium* increased with a higher use of irrigation water, from 500 mL to 1,000 -1,500 mL. Another study reported an increase in the content of phenolic compound (salidroside) under high VW conditions between 55 – 75% (Xinhai et al., 2003). Most studies report that water deficits can inhibit plant growth, possibly increasing the accumulation of SMs, such as the production of terpenoidphytoalexins (Nogues et al., 1998) and zealexins (Jaleel et al., 2007) in maize.

High MG levels were found in trees growing in soil with high Ca. This nutrient is needed for membrane permeability, helps in modulating responses to changing environments, and in plant defense mechanisms (Hepler, 2005; Lecourieux et al., 2006). Xu et al. (2021) reported that a higher soil VW (65% vs. 35% saturation) induced more Ca absorption in a perennial grass species (*Epichloe sinensis*), potentially increasing the solubility of Ca under higher soil moisture. Ca has been reported to either promote or suppress alkaloid production and accumulation. Poutaraud and Girardin (2005) reported that increasing soil Ca linearly increased the content of alkaloids in seeds of Meadow saffron (*Colchicum autumnale*), while the production of alkaloid isoquinoline in opium poppy (*Papaver somniferum*) was inhibited under high Ca levels (Bazuk and Lovkova, 1986). The bioavailability of soil nutrients is understood to be related with soil pH, with a slightly acidic to neutral soil (6.2 – 7.3) solubilizing ions, making them readily available for plant root uptake (Barber, 1995). However, pH within an acceptable range for bioavailability (pH=6.7 - 7.1) was reported to yield significantly lower alkaloid content in *Lupinus angustifolius* cultivars when compared with those grown at a lower pH (pH=5.3 - 5.8) (Jansen et al., 2012). Soils sampled in our study were highly acidic (pH less than 6) (**Figure 4**), which could also limit the availability of Ca and potentially lead to its deficiency. Even though we found higher content of MG in acidic soils (**Figure 4**), the influence of pH was found to be least important among the significant set of soil variables.

The actual synthesis and accumulation of various SMs is often induced and/or modulated through simultaneous interactions among a number of environmental factors (e.g., high irradiation is usually accompanied with elevated temperatures and water deficiency) (Uleberg et al., 2012; Virjamo et al., 2014; Wang et al., 2016; Arena et al., 2017; Ullrich et al., 2017; Woodrow et al., 2017). The complexity of biotic environmental interactions can also affect the production of SMs. For example, nicotine content in tobacco (*Nicotiana tobacum*) was reported to be higher in roots and leaves, resulting from a symbiotic presence of the arbuscular mycorrhizal fungi in the root system to ward off nematodes or leaf herbivores (Andrade et al., 2013). In the case of kratom, biotic stresses include grazing by herbivores such as Indianmeal moth (*Plodia interpunctella*), warehouse beetle (*Trogoderma variablile*), and khapra beetle (*Trogoderma granarium*) (Department of Agriculture, Bangkok, Thailand; www.dao.go.th), which can also directly affect the content of its alkaloids. The production of SMs, such as MG, would be an additional energy cost apart from that used in photosynthesis, which in turn could cause slower or stunted growth. Therefore, a tradeoff between growth and defense is chosen optimally, given the limited resources of energy within a tree (Iwasa, 2000). Kratom trees growing in different environmental conditions would have various defensive and adaptive mechanisms to deal with such micro-environments, including greater alkaloid production. Within the current analysis, we did not find any such interactions being significant among the environmental variables (abiotic stress only). This could be due to limited number of trees sampled and uncontrolled conditions in plants themselves as well as the environments. Interestingly, the differences in MG content of up to 72% and 74% were observed in leaves of kratom trees cultivated within close physical proximity to each other on two separate farms in Kedah and Penang, Malaysia, respectively (Chear et al., 2021). Variations in MG content were either due to genetic diversity, variations in production environment, or both. Although not quantified in their investigation, the influence of soil VW, pH, and Ca, as described in our study, may explain the variations in MG content reported by Chear et al. (2021). Therefore, we need to further investigate such synergistic effects of multiple environmental factors on plant secondary metabolism through a larger dataset in the future. Moreover, a more thorough investigation within controlled production environments is warranted to elucidate the suite of factors influencing the synthesis of MG and other important leaf alkaloids.

## Conclusion

The accumulation of alkaloids is influenced by a variety of environmental factors and for most plants, a change in an individual factor may alter the content of SMs even if other factors remain constant. We report the variability in the accumulation of MG found in kratom populations, growing in four regions of Thailand under different environmental conditions. The production of such alkaloids in terms of their extracted quantity is important for commercial purposes. As such, the optimized conditions for plant growth to accumulate a higher content of economically valuable alkaloids become important. We found that the MG content in all the sampled regions of Thailand was in a similar range to that reported by other studies in other parts of the world.

Key environmental variables correlated with high content of MG were high light intensity and VW (locations sampled in the C and S regions provided such conditions), while air RH, Ca, and soil pH were also found to be significant to a lesser degree. Relationships between environment and plant response, elucidated here, can be utilized under controlled conditions, such as a greenhouse, nursery, or plantation setting to obtain kratom leaves with similar MG levels found in the current commercial products. Further laboratory and greenhouse experiments are needed, however, to better understand the impact and interactions of these factors under controlled conditions.

## Data availability statement

The raw data supporting the conclusions of this article will be made available by the authors, without undue reservation.

## Author contributions

NL collected the data and wrote the manuscript, TA analyzed the data and wrote the manuscript with equal contribution and shares the first authorship, CN collected the data and identified species in the field and compared them with the herbarium specimens, SU collected the data and prepared the crude extract and determined the mitragynine content, PR and WS collected the data, RK, BP, CM, and AS reviewed the final manuscript. All authors contributed to the article and approved the submitted version.

## Funding

Thailand Science Research and Innovation (TSRI) through Kasetsart University Research and Development Institute (KURDI), Project No. FF(KU) 9.64.

## Acknowledgments

This work was financially supported by the Office of the Ministry of Higher Education, Science, Research and Innovation; and the Thailand Science Research and Innovation through the Kasetsart University Reinventing University Program 2021”. Under the funding project of Science, Research and Innovation Promotion Fund of the Thailand Science Research and Innovation (TSRI) through Kasetsart University Research and Development Institute (KURDI). Project No. FF(KU) 9.64.

## Conflict of interest

The authors declare that the research was conducted in the absence of any commercial or financial relationships that could be construed as a potential conflict of interest.

## Publisher’s note

All claims expressed in this article are solely those of the authors and do not necessarily represent those of their affiliated organizations, or those of the publisher, the editors and the reviewers. Any product that may be evaluated in this article, or claim that may be made by its manufacturer, is not guaranteed or endorsed by the publisher.
